# Melanotic Xp11 translocation renal cancer: report of a case with a unique intratumoral sarcoid-like reaction

**DOI:** 10.1186/1746-1596-9-81

**Published:** 2014-04-15

**Authors:** Lauren L Ritterhouse, Matthew D Cykowski, Lewis A Hassell, Gennady Slobodov, Barbara L Bane

**Affiliations:** 1 Department of Pathology, University of Oklahoma Health Sciences Center, BMSB 451 940 SL Young Blvd., Oklahoma City, OK 73104, USA; 2 Department of Pathology, Brigham and Women’s Hospital, Boston, MA, USA; 3 Department of Pathology and Genomic Medicine, Houston Methodist Hospital, Houston, TX, USA; 4 Department of Urology, University of Oklahoma Health Sciences Center, Oklahoma City, OK, USA

**Keywords:** Melanotic Xp11 translocation, TFE3, HMB-45, Renal cell carcinoma, Sarcoid-like reaction

## Abstract

**Background:**

Melanotic Xp11 translocation renal cancer is a rare tumor belonging to the family of microphthalmia-associated transcription factor (MiTF)/transcription factor E (TFE) neoplasms. This tumor family also includes alveolar soft part sarcoma, perivascular epithelioid cell neoplasms, Xp11 translocation renal cell carcinoma, and melanoma. To date, six confirmed melanotic Xp11 translocation cancers (five renal, one ovarian) have been reported in the literature.

**Case Report:**

Here, we report the clinical, histologic, immunohistochemical, and molecular features of a unique melanotic Xp11 translocation renal cancer arising in a 34-year-old African-American female. Histologically, the tumor was composed of epithelioid tumor cells arranged in a nested pattern. The cells had clear to eosinophilic granular cytoplasm, vesicular nuclear chromatin, and prominent nucleoli. Multifocal intracytoplasmic deposits of granular brown melanin pigment were identified and confirmed by Fontana-Masson stain. An unusual histologic feature, not previously reported in melanotic Xp11 translocation renal cancer, was a sarcoid-like granulomatous reaction consisting of tight epithelioid granulomas with lymphocytic cuffing, numerous giant cells, and calcifications. Nuclear transcription factor E3 expression was identified by immunohistochemistry and *TFE3* rearrangement was confirmed by fluorescence in situ hybridization. Additional immunohistochemical findings included immunoreactivity for HMB45, cathepsin K, and progesterone receptor; negative staining was seen with actin, desmin, cytokeratins, epithelial membrane antigen, CD10, vimentin, and PAX-8. The patient is currently free of disease, two years following initial clinicoradiologic presentation and twenty-two months following partial nephrectomy without additional therapy.

**Conclusion:**

This report further expands the spectrum of morphologic and clinical findings previously described in melanotic Xp11 translocation renal cancer, a distinctive tumor showing overlapping features between Xp11 translocation renal cell carcinoma, melanoma, and perivascular epithelioid cell neoplasms.

**Virtual slides:**

The virtual slide(s) for this article can be found here: http://www.diagnosticpathology.diagnomx.eu/vs/7225796341180634

## Background

Melanotic Xp11 translocation renal cancer (TRC) was first reported in 2009 as a neoplasm arising in the kidneys of two children with metastatic disease [1]. This initial report has been followed by reports of four additional melanotic Xp11 TRCs (three renal, one ovarian) with most occurring in young female patients [2-5]. Each of the reported tumors has demonstrated some degree of epithelioid cytomorphology, cytoplasmic melanin, and immunohistochemical positivity with markers of melanocytic differentiation (e.g., HMB-45, Melan-A, tyrosinase) and negativity with markers of epithelial (cytokeratin, epithelial membrane antigen) and muscle (desmin, smooth muscle actin) differentiation [2-5]. Critical to the diagnosis, all confirmed cases to date have demonstrated nuclear TFE3 protein expression by immunohistochemistry that reflects underlying *TFE3* rearrangement [1,5].

Based on the histologic and molecular features of melanotic Xp11 TRCs, Argani and colleagues considered these tumors unique members of the microphthalmia-associated transcription factor (MiTF)/Transcription Factor Enhancer (TFE) family of neoplasms [1]. The MiTF/TFE tumor family includes alveolar soft part sarcoma (ASPS), Xp11 translocation renal cell carcinoma (RCC), t(6;11) RCC, perivascular epithelioid cell tumor (PEComa), melanoma, and clear cell sarcoma of soft tissues [1]. The MiTF/TFE neoplasms have in common the overexpression of MiTF and/or TFE3 or TFEB transcription factors - a subfamily in the helix-loop-helix family of transcription factors [1,2]. These tumors may also have overlapping histologic features. Thus, Argani and colleagues have proposed that melanotic Xp11 TRCs demonstrate features seen in ASPS, PEComa, Xp11 RCC, and melanoma [1]. However, melanotic XP11 TRCs are separated from these tumors by their combination of melanin pigmentation, immunohistochemical negativity for S-100 and markers of epithelial and muscle differentiation, and most critically, rearrangement of *TFE3*[1,2].

Herein, we present a unique case of a melanotic Xp11 TRC occurring in a 34-year old African American female. Unique features included a sarcoid-like reaction that consisted of a lymphocytic and granulomatous infiltrate with numerous giant cells and calcifications. Rare descriptions of similar histologic phenomena exist for conventional RCC [6-10], but have not previously been reported as part of the morphologic spectrum of melanotic Xp11 TRC.

## Case presentation

### Clinical and radiologic findings

A 34-year-old African American female presented with a 6-month history of dull left-sided flank pain (probably not attributable to the subsequently discovered renal mass per the urology team). The patient’s medical history was significant for obesity, hypertension, and tobacco use. There were no other known environmental risk factors. Family history was significant for pancreatic cancer (mother), leukemia (father), and breast cancer (two maternal aunts who died in their thirties). The patient had no personal or family history of tuberous sclerosis.

Clinical examination was notable for an ill-appearing, obese patient with mild left costovertebral angle tenderness. Computed tomography (CT) scans demonstrated an endophytic 5.4 cm mass within the anterior middle and lower poles of the left kidney. Avid contrast enhancement and areas of internal low-attenuation were identified within the mass and a radiologic impression of RCC was offered. The patient subsequently underwent open partial left nephrectomy.

### Pathologic findings

On gross examination the specimen had an intact renal capsule and an encapsulated, bulging, yellow-tan, cortical tumor measuring 4.8 × 4.6 × 4.0 cm. The tumor showed patchy areas of dark-brown to dark-red pigmentation and focal necrosis. There was no evidence of extracapsular extension. Surgical margins were grossly free of tumor.

**Figure 1:**
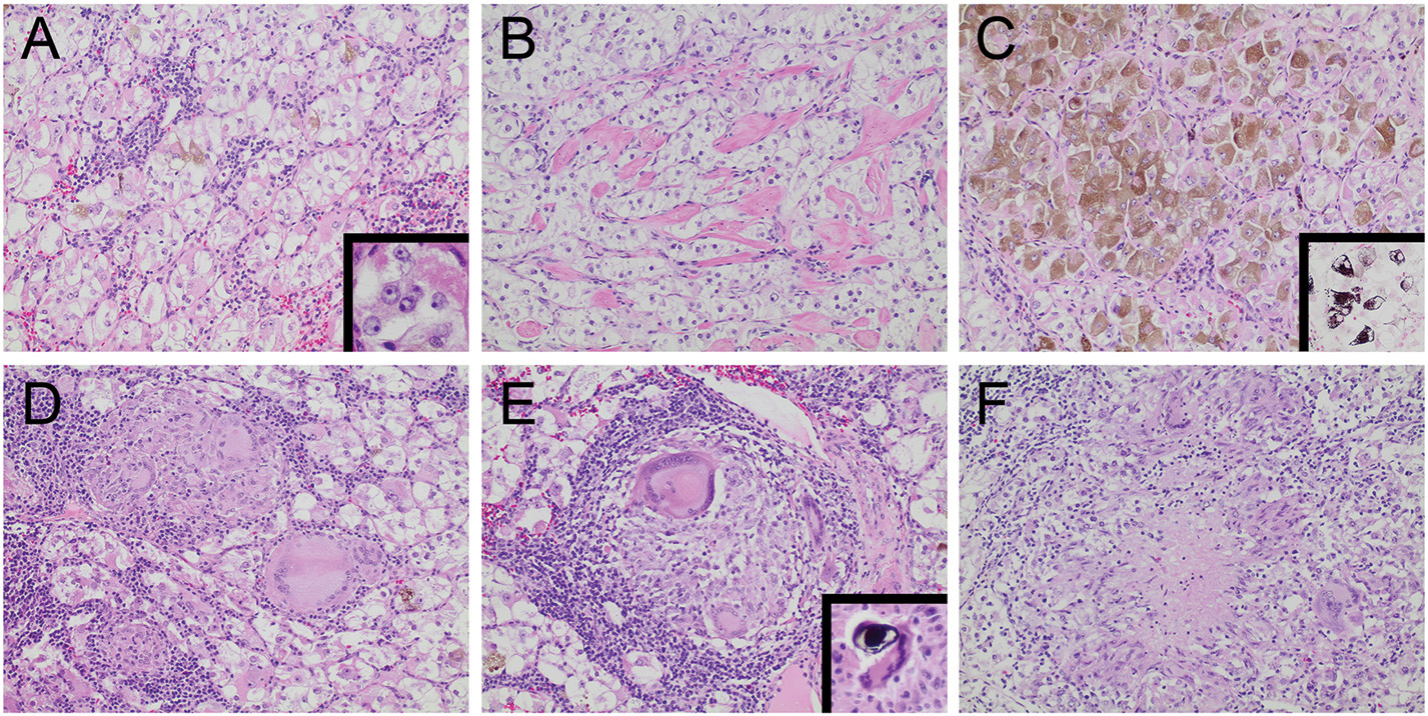
**Microscopic features.** A nested growth pattern of epithelioid tumor cells with clear to lightly eosinophilic cytoplasm, prominent cell membranes, and interstitial lymphocytic infiltrate was characteristic **(A)**, and occasional thick collagenous septa were present **(B)**. At high-power, slightly irregular nuclear contours, vesicular chromatin, and prominent nucleoli were apparent **(A, inset)**. Patchy, fine to slightly coarse cytoplasmic melanin **(C)** was confirmed histochemically with a Fontana-Masson silver stain (**C**, inset). Unique to this case were intratumoral sarcoid-like and granulomatous inflammatory reactions, including tightly formed, noncaseating epithelioid granulomas with conspicuous multinucleated giant cells and a lymphocytic cuff **(D, E, F)**. Calcifications were prominent within foci of granulomatous inflammation. Focally these calcifications had a shell-like appearance as seen in Schaumann (conchoidal) bodies (**E**, inset).

Routine H&E sections of tumor demonstrated a nested arrangement of epithelioid cells with clear-to-eosinophilic granular cytoplasm (Figure 1A, B). Patchy, granular, brown pigment was identified (Figure 1A, C) that stained by Fontana-Masson technique (Figure 1C and inset). Nuclei were slightly irregular with vesicular chromatin and evident nucleoli (Figure 1A, inset). Noncaseating, sarcoid-like epithelioid granulomas with an accompanying lymphocytic infiltrate and numerous giant cells were frequent (Figure 1D, E). Giant cells were often associated with psammomatous calcifications and occasional calcifications with the appearance of Schaumann (conchoidal) bodies (Figure 1E, inset). Rare foci with a palisading histiocytic infiltrate and a central core of necrobiotic material were also seen (Figure 1F). Giant cells also focally contained refractile, nonpolarizable foreign material.

**Figure 2:**
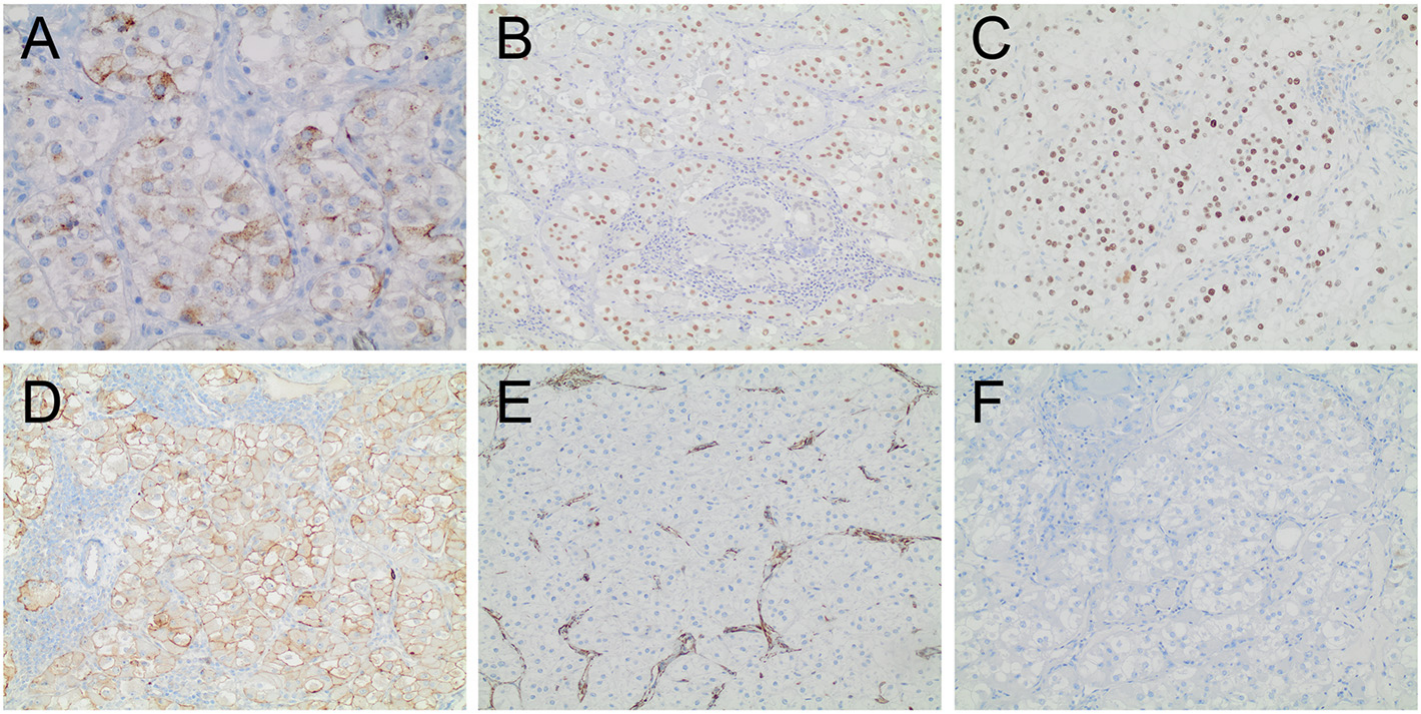
**Immunohistochemical features.** The tumor showed focal, strong reactivity with HMB-45 **(A)**, diffuse, strong nuclear reactivity with TFE3 **(B)**, patchy moderate immunoreactivity with progesterone **(C)**, and strong membranous positivity with CD117 **(D)**. Pertinent negative stains included vimentin **(E)** and pan-cytokeratin **(F)**.

The immunohistochemical features of the tumor are demonstrated in Figure 2 and listed in Table 1. The tumor cells showed patchy immunoreactivity for HMB-45 in a cytoplasmic and membranous pattern (Figure 2A), patchy moderate immunoreactivity for progesterone receptor (Figure 2B), diffuse nuclear staining for TFE3 (Figure 2C) and patchy but strong, membranous positivity with C-kit/CD117 (Figure 2D). Cathepsin K and focal E-cadherin staining were also seen. Tumor cells were negative for all epithelial and renal tubule markers including pan-cytokeratin (CK), epithelial membrane antigen, BerEP-4, CD10, CK34βE12, CK8/18, CK7, and carbonic anhydrase IX. Tumor cells were also negative for markers of muscle differentiation including smooth muscle actin, muscle specific actin, and desmin and negative for vimentin and PAX-8 (Figure 2E, F). Periodic acid-Schiff stain demonstrated abundant, diastase-susceptible glycogen within tumor cells. Prussian blue demonstrated stainable iron predominantly within giant cells in foci of granulomatous inflammation and calcification.

**Table 1:** Features of seven melanotic Xp11 translocation renal cancers reported in the literature

**Year/Author**	**Patient information**	**Clinical presentation**	**Radiologic findings**	**Pertinent IHC results**	**Genetic features**	**Therapy/Outcome**
**2009, Argani *et al.***	11-year-old black/Haitian male	Presumptive diagnosis of WT in native country 2 years prior	L renal tumor (21.5 cm), BL lung nodules, enlarged mesenteric nodes	**Pos**: HMB45, TFE3, Melan-A, **Neg**: Desm, Actin, RCC, EMA, S100, Pan-CK, MiTF, PAX-8	*TFE3*, BA FISH signal (unbalanced)	Nephrectomy; catheter placed for CT; **outcome *NS***
**2009, Argani *et al.***	12-year-old female, race/ethnicity *NS*	Sudden onset of L flank pain, hematuria	Large L renal tumor, mid/upper poles, enlarged RP, mesenteric nodes	**Pos**: HMB45, TFE3, tyrosinase, Melan-A, **Neg**: Desm, Actin, RCC, EMA, S100, CK, MiTF, CD99, Vim, PAX-8	*TFE3*, BA FISH signal (balanced)	Biopsies; CT; **Dead of disease at 9 months**
**2009, Chang *et al.***	18-year-old Taiwanese female	Dizziness and epigastric pain	10 cm R renal tumor	**Pos**: HMB45, TFE3, **Neg**: Desm, SMA, Vim, EMA, S100, Pan-CK, CD117, MiTF	*PSF-TFE3* gene fusion; *TYRP1, TYR, MiTF* upregulation	Nephro-ureterectomy, LND; pT2; **disease free at 3 months**
**2011, Varinot *et al.***	30-year-old North African female	Weakness, dyspnea, L arm paresthesia	12.5 cm renal tumor with IVC and PA thrombus	**Pos**: HMB45, TFE3, Melan-A, **Neg**: Desm, SMA, Pan-CK, S100, PAX-8	*TFE3*, BA FISH signal	Nephrectomy; therapy *NS*; **widely metastatic disease**
**2012, LeGallo *et al.***	15-year-old African-American female	1-day h/o severe abdominal pain	14 cm R ovary tumor, with IVC compression and bowel displacement	**Pos**: HMB45, tyrosinase, **Neg**: Desm, SMA, S100, EMA, Pan-CK, CD117	*TFE3*, BA FISH signal; *EWSR1* trisomy	Ovarian mass excision; no CT/RT; **disease free at 9 months**
**2013, Rao *et al.***	14-year-old female, race/ethnicity *NS*	*NS*	*NS*	**Pos**: TFE3, CathK, **Neg**: *NS*	*TFE3*, BA FISH signal	Procedure *NS*; pT1N0Mx; **further outcome *NS***
**Present Case**	34-year-old African-American female	6-month h/o dull L flank pain	4.6 cm midpole of L kidney tumor	**Pos**: HMB45, TFE3, CD117, PR, CathK, **Neg**: Desm, SMA, ER, Pan-CK, CK7, EMA, Vim, PAX-8	*TFE3*, BA FISH signal	Partial nephrectomy; pT1b; no CT/RT; **disease free at 22 months**

*Abbreviations*: *BA* break apart, *BL* bilateral, *CathK* cahepsin K, *CK* cytokeratin, *CT* chemotherapy, *Desm* Desmin, *EMA* epithelial membrane antigen, *ER* estrogen receptor, *EWSR1* Ewing sarcoma breakpoint region 1, *FISH* fluorescence in-situ hybridization, *h/o* history of, *IVC* inferior vena cava, *L* left, *LND* lymph node dissection, *MiTF* microphthalmia-associated transcription factor, *NS* not specified, *PA* pulmonary artery, *PR* progesterone receptor, *R* right, *RP* retroperitoneal, *RT* radiotherapy, *SMA* smooth muscle actin, *TYR* tyrosinase, *TYRP1* tyrosinase-related protein 1, *Vim* vimentin, *WT* Wilms tumor.

### Molecular findings

Break-apart FISH analysis showed evidence of *TFE3* rearrangement with 60% of cells displaying a split signal (study performed by Dr. Pedram Argani and Johns Hopkins Reference Laboratories).

## Conclusions

We describe a case of melanotic Xp11 TRC arising in a 34-year old African American female, unique for a sarcoid-like histologic reaction within the tumor stroma. To our knowledge, this is the seventh report of this entity, the oldest patient thus far reported, and the first to describe this peculiar pattern of lymphocytic and granulomatous inflammation. This “sarcoid-like” pattern comprised compact, noncaseating epithelioid granulomas with prominent lymphocytic cuffing, numerous giant cells, and psammomatous microcalcifications (Figure 1D-F). An additional unique feature of this case was its occurrence in an adult patient. In contrast, five of the previous six confirmed cases of melanotic Xp11 TRCs were in patients aged 18 years or younger [1-3].

The present case demonstrated other clinical and histologic features previously described in melanotic Xp11 TRC, including presenting symptoms of abdominal pain and female predisposition (all but one reported case to date occurring in females). Also observed, as with other cases providing complete demographic data, the tumor reported herein occurred in a black/African-American patient. This demographic association has also been reported in four of the five cases with complete data as demonstrated in Table 1 (the fifth case occurring in a Taiwanese female). Histologically, features identified here and reported previously include tumor cells with epithelioid cytology and clear to eosinophilic cytoplasm, a nested growth pattern, and cytoplasmic melanin. Immunohistochemical positivity with melanocytic markers (HBM-45, tyrosinase, Melan-A), TFE3, and cathepsin K is an additional common finding across this and previously reported cases (see Table 1). As in previous cases, the tumor described here demonstrated diffuse TFE3 nuclear staining and an underlying *TFE3* rearrangement was confirmed by break-apart FISH assay. In addition, almost all melanotic Xp11 TRCs have demonstrated negativity with markers of muscle differentiation (desmin, actin, caldesmon), epithelial and renal tubule markers (CD10, EMA, cytokeratins, carbonic anhydrase IX), and various additional markers such as MiTF, S-100, and PAX-8 (see Table 1). Two novel immunohistochemical results here were positivity with progesterone receptor (estrogen receptor was negative) and CD117 (C-kit), which is typically reported as negative. Studies of progesterone receptor and CD117 in additional melanotic XP11 TRCs will be required to explore the possible significance of these unique immunohistochemical findings.

The major differential diagnosis of melanotic Xp11 TRC includes conventional clear cell RCC, papillary RCC, translocation renal cell carcinomas (Xp11 RCC and t(6;11)), RCC unclassified, melanoma, and PEComa, including angiomyolipoma (AML) and its epithelioid variant. As described above, immunohistochemical negativity with all epithelial and renal tubule markers, S-100, desmin and actin eliminates many of these other diagnostic possibilities. Further differentiating them from renal epithelial tumors, melanotic Xp11 cancers are unique in their lack of reactivity with PAX-2 and PAX-8 [1]. Immunohistochemical reactivity with HMB-45 and Melan-A, however, can also be seen in PEComa/AML, t(6;11) translocation carcinomas, and clear cell sarcoma [1,11,12]. Immunohistochemical studies for PAX-2 and CD10, both often positive in translocation RCCs, may prove useful in making this distinction as these stains are uniformly negative to date in melanotic Xp11 TRCs (Table 1). The immunohistochemical marker MiTF also may contribute to this differential, as it is frequently positive in melanoma, clear cell sarcoma, and PEComa, but is typically negative in melanotic Xp11 TRC [1].

The critical studies in the diagnosis of melanotic Xp11 TRCs remain the demonstration of TFE3 nuclear expression and underlying *TFE3* rearrangement. TFE3 expression is also seen in alveolar soft part sarcoma, melanotic Xp11 TRCs, Xp11 RCC, and rare PEComas that are negative for MiTF [1,13,14]. Notably, these other three histologically similar, TFE3 positive tumors do not typically contain cytoplasmic melanin. Nonetheless, FISH and/or other molecular studies (such as reverse transcription polymerase chain reaction) are still recommended to rule out gene fusions diagnostic of these other entities. One caveat of molecular verification is that Chang and colleagues identified a *PSF-TFE3* fusion in melanotic Xp11 TRC that is also reported in Xp11 RCC [2]. This raises the possibility that melanotic Xp11 TRC and Xp11RCC are closely related, although in the majority of cases to date the gene fusion partner of *TFE3* has not been described [2]. *TFE3*-rearranged PEComas that exhibit strong TFE positivity and minimal desmin and actin positivity have also recently been described [14]. Therefore, *TFE3*-rearranged PEComa should be carefully considered as a diagnostic possibility in tumors with histologic features that overlap with melanotic Xp11 TRC.

The sarcoid-like reaction described in this tumor has been reported in conventional RCC [6-10] and other malignancies [15-17], but has not previously been reported in melanotic Xp11 TRC. Non-neoplastic conditions associated with granulomatous inflammation, with and without necrosis, include infections (mycobacterial, fungal, parasitic), various autoimmune conditions (e.g., biliary cirrhosis, ankylosing spondylitis, and rheumatoid arthritis) [18], hyperprolactinemia [19], chemical exposures and foreign body-type reactions [6]. To our knowledge, our patient had no history of these conditions and no prior biopsy diagnosis of granulomatous inflammation at any site. Thus the findings appear to be tumor specific. However, the pathogenesis and significance of these findings in this case, as well as in prior tumors, are not known. A similar sarcoid-like reaction was recently reported in the RCC of a 62-year-old Caucasian patient with no history of sarcoidosis [6]. In contrast to our case, that study reported the granulomatous inflammation in the peritumoral region, but not within the tumor. It has been postulated that this process is the result of a T-cell mediated host response to antigens expressed or produced by tumor cells [6,20]. In other instances, similar reactions have been reported following chemical exposure such as the use of contrast media in imaging studies [8]. Similar sarcoid-like tumor reactions have been shown to have positive prognostic significance in patients with Hodgkin disease and gastric cancer [16,17]. Their significance here and in conventional RCC remains unknown, but deserving of further investigation.

An important future direction will be to investigate the histologic and/or genetic features of these tumors that predispose to indolent or frankly malignant behavior. As demonstrated in Table 1, of the five patients with reported outcome data available, two patients presented with widely disseminated metastatic disease. One of these two patients was dead of disease within nine months of diagnosis despite chemotherapy. The remaining patients, including this case, were free of disease at 3, 9, and 22 months, respectively. There is no clear reason at this time for this significant discrepancy in metastatic potential and clinical outcome. Further investigation of the underlying *TFE3* rearrangements is warranted. The gene fusion partners are rarely known (with the exception of the report by Chang and colleagues [2]), and this may provide valuable genetic information that relates to prognosis. Additional studies of melanotic Xp11 TRCs will be important to further define the prognostic implications and appropriate therapies for these rare tumors. Correct classification of these distinct neoplasms is important as the MiTF/TFE3-related cellular pathways may eventually provide targets for novel therapeutic agents.

### Consent

Written informed consent was obtained from the patient for publication of this Case Report and any accompanying images. A copy of the written consent is available for review by the Editor-in-Chief of this journal.

## Abbreviations

ASPS: Alveolar soft part sarcoma; MiTF: Microphthalmia-associated transcription factor; PEComa: Perivascular epithelioid cell tumor; RCC: Renal cell carcinoma; TFE: Transcription factor E; TRC: Translocation renal cancer.

## Competing interests

The authors declare that they have no competing interests.

## Authors’ contributions

LR reviewed the data and prepared the initial manuscript draft. MC, BB, GS, and LH participated in the initial work-up of the case described herein, reviewed the data, and assisted LR in preparing the final version of the manuscript. All authors read and approved the final manuscript.

## Authors’ information

LR is pathology resident at Brigham and Women’s Hospital and MC is a neuropathology fellow at Houston Methodist Hospital. BB is the team leader of urologic pathology and LH is the director of anatomic pathology in the Department of Pathology, University of Oklahoma Health Sciences Center. GS is chief of the section of Female Pelvic Medicine and Reconstructive Surgery in the Department of Urology, University of Oklahoma Health Sciences Center.
